# Endometrial stem cells alleviate cisplatin-induced ferroptosis of granulosa cells by regulating Nrf2 expression

**DOI:** 10.1186/s12958-024-01208-8

**Published:** 2024-04-11

**Authors:** Rumeng Pan, Rongli Wang, Feiyan Cheng, Lihui Wang, Zhiwei Cui, Jing She, Xinyuan Yang

**Affiliations:** Department of Obstetrics and Gynecology, The First Affiliated Hospital of Xi’an Jiaotong University, 710061 Xi’an, China

**Keywords:** Ferroptosis, Premature ovarian failure, Endometrial stem cells, Nrf2, Ferrostatin-1

## Abstract

**Background:**

Premature ovarian failure (POF) caused by cisplatin is a severe and intractable sequela for young women with cancer who received chemotherapy. Cisplatin causes the dysfunction of granulosa cells and mainly leads to but is not limited to its apoptosis and autophagy. Ferroptosis has been also reported to participate, while little is known about it. Our previous experiment has demonstrated that endometrial stem cells (EnSCs) can repair cisplatin-injured granulosa cells. However, it is still unclear whether EnSCs can play a repair role by acting on ferroptosis.

**Methods:**

Western blotting and quantitative reverse-transcription polymerase chain reaction (qRT-PCR) were applied to detect the expression levels of ferroptosis-related genes. CCK-8 and 5-Ethynyl-2’-deoxyuridine (EdU) assays were used to evaluate cell viability. Transmission electron microscopy (TEM) was performed to detect ferroptosis in morphology. And the extent of ferroptosis was assessed by ROS, GPx, GSSG and MDA indicators. In vivo, ovarian morphology was presented by HE staining and the protein expression in ovarian tissue was detected by immunohistochemistry.

**Results:**

Our results showed that ferroptosis could occur in cisplatin-injured granulosa cells. Ferroptosis inhibitor ferrostatin-1 (Fer-1) and EnSCs partly restored cell viability and mitigated the damage of cisplatin to granulosa cells by inhibiting ferroptosis. Moreover, the repair potential of EnSCs can be markedly blocked by ML385.

**Conclusion:**

Our study demonstrated that cisplatin could induce ferroptosis in granulosa cells, while EnSCs could inhibit ferroptosis and thus exert repair effects on the cisplatin-induced injury model both in vivo and in vitro. Meanwhile, Nrf2 was validated to participate in this regulatory process and played an essential role.

**Supplementary Information:**

The online version contains supplementary material available at 10.1186/s12958-024-01208-8.

## Background

Premature ovarian failure (POF) is a gynecological endocrine disease that forces many women of reproductive age to face physical and mental stress. Data shows that the global incidence rate of premature ovarian failure has reached 1% in women under 40 [1]. Currently, the high incidence and the trend of the younger age of tumors make more and more young women with cancer face the occurrence of iatrogenic premature ovarian failure. Numerous experiments have demonstrated that chemotherapy can severely impair ovarian reserve and follicle development [2, 3]. Cisplatin, as one of the conventional chemotherapy drugs in clinics, has been proven to have reproductive toxicity under long-term and high-dose exposure, which can lead to POF [4, 5].

How to improve the fertility and life quality of women with POF has always been a delicate problem that received much attention in recent years. The outstanding progress in regenerative medicine has made mesenchymal stem cells (MSCs) a hot spot for research, and the POF field is no exception [6]. Although our previous study has indicated that endometrial stem cells (EnSCs), a type of MSCs that can be acquired by noninvasive repeated sampling, have repair effects on the cisplatin-induced POF animal model [7], there is spacious room to explore its specific repair mechanism.

Accumulating evidence suggests that dysfunction of ovarian granulosa cells, which is closely associated with the development of oocytes, is an important enabler of POF. Most of the current studies have elaborated that the occurrence of POF is closely related to the apoptosis of granulosa cells and the atresia of follicles [8, 9]. However, there is a lack of experimental data about whether other forms of cell death are also involved and play an unknown but important role during the occurrence and development of POF.

Ferroptosis is a unique type of iron-dependent programmed death first proposed in 2012 by Dixon et al., which is distinct from apoptosis and characterized by reactive oxygen species production and lipid peroxidation [10]. Studies have manifested that ferroptosis is tightly connected with various diseases [11, 12], including reproductive endocrine-related diseases such as polycystic ovary syndrome (PCOS) [13, 14] and POF [15–18]. A previous study has suggested a correlation between ferroptosis and oocyte loss during the primordial follicle assembly phase by single-nucleus RNA sequencing (snRNA-seq) of mouse ovarian cells exposed to cigarette smoke [19]. Wang et al. found BNC1 deficiency-induced primary ovarian insufficiency is based on oocyte ferroptosis via the NF2-YAP pathway [15]. Furthermore, POF is not only associated with the ferroptosis of oocytes. Zhao et al. revealed that sphingosine 1-phosphate (S1P) can alleviate radiation-induced ferroptosis in KGN cells by upregulating GPX4 expressions to restore ovarian function [20]. In addition, a previous study indicated cisplatin-induced ferroptosis mediated premature ovarian insufficiency in a rat model, and ferroptosis inhibitor Vitamin E reversed the reproductive toxic effects of cisplatin on the ovary [16]. As noted above, increasing evidence suggests that ferroptosis may contribute to the occurrence and development of POF. However, research on the relationship between ferroptosis and POF is still insufficient and deserves further exploration.

Nuclear factor erythroid 2-related factor 2 (Nrf2/NFE2L2), a prevalent anti-oxidative stress regulatory transcription factor, caught our attention. In the D-Galactose induced-premature ovarian failure mice model, daphnetin can attenuate POF by upregulating Nrf2 expression [21]. Meanwhile, Ding et al. found that Nrf2 activation also participated in the repair process of human placental mesenchymal stem cells to POF [22]. Therefore, we speculated that Nrf2 may play an essential regulatory role in the repair process of EnSCs to POF. Moreover, Nrf2 has been widely considered one of the potent regulators of ferroptosis [23, 24]. However, little is known about whether EnSCs can mitigate ferroptosis and restore ovarian function by acting on Nrf2 .

Despite the increasing attention MSCs drew and the wide repair effects on POF already discovered, the exact mechanism is poorly understood. Besides, ferroptosis is explored in many diseases but is rarely reported in POF. Here, we unveil that EnSCs can inhibit the ferroptosis of granulosa cells to achieve a repair effect and explore the possible underlying mechanisms. Our study implies that ferroptosis promotes POF and reinforces our opinion that EnSCs have considerable potential to treat POF. It is beneficial to develop a novel strategy to protect against ovarian damage by inhibiting the ferroptosis of granulosa cells. And our findings further complement the mechanism of stem cell therapy for POF.

## Methods

### Cell culture

The human ovarian granulosa cell line KGN acquired from Procell Life Science & Technology Co., Ltd (Procell CL-0603, China) was cultured in DMEM/F12 medium (Hyclone, USA) with 10% fetal bovine serum (FBS, Sijiqing, China) in an incubator at 37℃ available with 5% CO_2_. The medium was replaced as needed. To maintain an exponential growth phase during the experiments, when cells reached more than 90% confluence, they were separated using 0.25% trypsin-EDTA and passaged.

The extraction of endometrial stem cells and establishment of EnSCs-injured granulosa cells co-culture model were detailedly elaborated on in our previous study [25]. After the approval of the Ethical Committee of the First Affiliated Hospital of Xi’an Jiaotong University and the subscription of written informed consent, menstrual blood samples (approximately 10 ml each) were collected from six healthy women aged between 25 and 30 years old on the first day of menstruation. The samples were then transferred into a 50 ml centrifuge tube containing 10 ml of phosphate-buffered saline (PBS), penicillin (100 U/ml), streptomycin (100 mg/ml), 0.25 mg/ml amphotericin B, and 2 mM ethylenediaminetetraacetic acid (EDTA). Next, according to the manufacturer’s instructions, endometrial stem cells were separated and purified using Ficoll-Paque Plus (GE Healthcare, Amersham, UK). The cells were suspended in DMEM/F12 supplemented with 10% fetal bovine serum (Sijiqing, China), streptomycin (100 mg/ml), and penicillin (100 U/ml) and then cultured in a humidified incubator at 37℃ in 5% CO_2_. The cell culture medium was changed every three days. When the cells reached 90% confluence, they were detached using 0.25% trypsin–EDTA and passaged at 1:3.

Firstly, we seeded KGN cells with appropriate density in a six-well plate. After 24 h, 10 µM cisplatin was added and then incubated for 48 h to acquire damaged granulosa cells in the lower chamber. Subsequently, 6 × 10^5^ EnSCs were seeded in the upper chamber using a 6-well transwell insert ( 6.5 mm polycarbonate membrane, 4.0 μm pore size, Corning, USA ). The cells in the lower chamber were harvested after being co-cultured in a 5% CO_2_ incubator at 37℃ for 72 h.

### Reagents

Reagents used in this study were as follows: Cisplatin (232,120, Sigma-Aldrich, USA) was prepared in normal saline. Ferrostatin-1 (HY-100,579, MedChemExpress, China) and ML385 (HY-100,523, MedChemExpress, China) were dissolved in dimethyl sulfoxide (DMSO, 276,855, Sigma-Aldrich, USA).

### Cell counting kit 8 (CCK8) assay

Cell counting kit 8 (CCK8) assay was conducted to evaluate cell viability. KGN cells were planted into 96-well plates and then treated with different treatments according to experimental purposes. Then cells were incubated with contained 10% CCK8 reagent (AC0011S, AccuRef Scientific, China) complete medium for 2 h at 37℃ with 5% CO_2_. Finally, the microplate reader (BioTek Epoch2, America) was used to measure the absorbance of samples at 450 nm to determine the cell viability.

### Ethynyl-2-deoxyuridine (EdU) assay

EdU assay was performed by using BeyoClick™ EdU Cell Proliferation Kit with Alexa Fluor 594 (Beyotime Biotechnology, China). The treated cells were incubated with 10 µM EdU for 2 h. After the completion of EdU labeling cells, cells were slightly washed with phosphate buffer saline (PBS), and then 4% paraformaldehyde was added to fix for 30 min at room temperature. Subsequently, cells were permeabilized with 0.5% Triton X-100 for 15 min after washing with PBS three times. Removing the washing solution in the previous step, the prepared click reaction solution was put into each well and then incubated in the dark for 30 min at room temperature. Then the click reaction solution was removed and washed three times with PBS, 5 min each time. Next, nuclei were stained using Hoechst 33,342 in the dark for 10 min and washed as before. Finally, fluorescence image was detected using a fluorescent inverted Leica DMIRB microscope (Leica, Germany).

### Western blotting

Cells were collected and used to extract the total protein with the mixture of the radioimmunoprecipitation (RIPA) lysis buffer, the protease inhibitor cocktail (1:50) and phenylmethanesulfonyl fluoride (PMSF) (1:100). The protein concentrations were quantified using a Bradford Protein Assay Kit (Proandy, China). The same amount of protein (30 µg) was separated by 10–12% SDS-PAGE electrophoresis followed by transferring onto the polyvinylidene difluoride (PVDF) membranes. Then the membranes were blocked in 5% non-fat skim milk at room temperature for 1 h. After three times washes with Tris-HCl buffered saline with Tween-20 (TBST), these membranes were put into the corresponding primary antibodies and incubated overnight at 4 ℃. The primary antibodies included GPX4 (1:1000, T56959, Abmart, China), Nrf2 (1:3000, 16396-1-AP, Proteintech, China), FTH1 (1:1000, PTM-6761, PTMBio, China), SLC7A11 (1:1000, T57046, Abmart, China), GAPDH (1:5000,10494-1-AP, Proteintech, China). The next day, the membranes were washed with TBST and put into the HRP-conjugated secondary antibody (1:5000, SA00001-2, Proteintech, China) at room temperature for another 1 h. Finally, the images could be captured by enhanced chemiluminescence reagent (ECL, Beyotime Biotechnology, China) and chemiluminescent imager (Tanon-5200, China).

### RNA extraction and quantitative real time-polymerase chain reaction (qRT-PCR)

Trizol reagent (Invitrogen, USA) was applied to extract the total RNA, and then RNA was reverse-transcribed to cDNA under the condition of purity and concentration up to standard. qRT-PCR was conducted using 2x Fast qPCR Master Mixture (DN2055-05, DiNing, China) on BioRad CFX manager quantitative fluorescence PCR instrument. The 2^−ΔΔCt^ method was used to analyze the target genes’ relative expression levels. The primer sequences used in this study were provided in Table S1.

### Measurement of intracellular ROS

According to the instruction of Reactive Oxygen Species Assay Kit (S0033S, Beyotime Biotechnology, China), cells were first treated accordingly, digested and collected, and then 2’, 7’-dichlorofluorescein-diacetate (DCFH-DA) with a final concentration of 10 µM diluted with the serum-free medium was added to suspend the cells, and the cells were sheltered from light at 37℃ and incubated for 30 min. Centrifuge tubes were mixed upside down every 5 min to make the probe fully contact with the cells. Then, cells were cleaned three times with a serum-free cell culture medium to adequately remove extra DCFH-DA. Finally, the intracellular ROS content was assessed by the optical density (OD) value which was measured by a microtiter plate reader (BioTek Cytation 5M, America) at 488 nm excitation wavelength and 525 nm emission wavelength.

### GPx activity assay

According to the instructions of the corresponding kits, the activities of glutathione peroxidase (GPx, S0056) were analyzed by specific commercial assay kits (Beyotime Biotechnology, China).

After the treated fresh cells were collected and lysed, the supernatant was taken to detect GPx. GPx activity was detected following the manufacturer’s protocol. The absorbance at 340 nm was recorded every 1 min for 5 consecutive minutes to obtain 6 points of data in the microplate reader (BioTek Epoch2, America) at 25℃. Finally, the experimental results were acquired according to the calculation method in the manufacturer’s protocol.

### GSSG detection

The changes in oxidized glutathione content after different treatments were detected by GSH and GSSG Assay Kit (S0053, Beyotime Biotechnology, China). In brief, the treated fresh cells were washed with PBS and centrifuged, and the supernatant was removed to retain cell precipitation. Add a protein removal reagent M solution with a cell precipitation volume of 3 times. Then, the samples were subjected to two rapid freeze-thaw cycles. Next, they were placed in an ice bath for 5 min and centrifuged at 4℃, 10,000 g for 10 min. The supernatant was collected and then mixed with the detection reagent of the kit according to the specifications. The absorbance at 412 nm was recorded using a microplate reader (BioTek Epoch2, America) and the total protein content was determined using the Bradford Protein Assay Kit (Proandy, China).

### Lipid peroxidation MDA assessment

The determination of MDA has been widely used as an indicator of lipid oxidation. After treatment, the fresh cells were collected and lysed to acquire the supernatant to detect MDA.

MDA detection working solution was prepared according to the instructions. After mixing the samples and the working solution, the samples were heated at 100℃ for 15 min. Then, samples were cooled to room temperature in a water bath and centrifuged at 1000 g at room temperature for 10 min. Finally, 200 µL supernatant was added to 96-well plates and the absorbance was measured at 532 nm using a microplate reader (BioTek Epoch2, America).

### Transmission electron microscopy (TEM)

Cells were treated with 10 µM cisplatin for 24 h and then collected. 2.5% glutaraldehyde special fixative for electron microscopy was slowly added to fix and 1% OsO4 was used to postfix. Then the slices of these fixed cells were dehydrated in ethanol and embedded before cutting ultrathin sections. After being stained with uranyl acetate and lead citrate, these sections were visualized by transmission electron microscopy (H-7650, Hitachi, Japan).

### POF animal model establishment

C57BL/6 female mice aged 6–8 weeks old purchased from the Animal Center of Xi’an Jiaotong University were used to establish the POF model. The experimental protocol was approved by the Ethical Committee and the Institutional Animal Care and Use Committee of Xi’an Jiaotong University (protocol code: 2022 − 190, date: 4 March 2022). Before the experiments, mice were kept untreated for one week for environmental adaptation. And then they were randomly divided into six groups, and each group had five mice. According to our previous protocol [7], the POF model was established by intraperitoneal injection of cisplatin (2 mg/kg/d) into mice for 7 consecutive days.

### EnSCs transplantation and Fer-1 administration

After the POF model was successfully established, the EnSCs transplantation group was constructed from day 8 onwards by tail vein injection of EnSCs (passage 3–5, 2 × 10^6^ in 200 µl cell suspensions) (Fig. 4D). At the same time, the control group and the cisplatin group were administered with an equal volume of the medium.

On the other hand, ferrostatin-1 (5 mg/kg) was intraperitoneally administered 1 h earlier than cisplatin for 7 consecutive days as the cisplatin + Ferrostatin-1 group (Fig. S1A). The solvent for ferrostatin-1 in vivo was 0.1% DMSO. An equal volume of DMSO was used in the control group and cisplatin group. After all processing was completed, blood samples were collected from the mice’s eyeballs. Then they were euthanized by a dislocated cervical spine and bilateral ovaries of mice were harvested.

### Measurement of total antioxidant capacity of serum

Mice serum was collected after different treatments. The total antioxidant capacity (T-AOC) of serum was measured by the corresponding assay kit (S0116, Beyotime Biotechnology, China). The principle is that the antioxidant can reduce Fe^3 +^ -TPTZ (tripyridyltriazine) to produce blue Fe^2 +^ -TPTZ under acidic conditions, and then the total antioxidant capacity of the sample can be obtained by measuring blue Fe^2 +^ -TPTZ at 593 nm. A higher absorbance represents a stronger antioxidant capacity.

### Hematoxylin-eosin staining

The collected ovarian tissues were immersed in 4% paraformaldehyde solution for fixation and subsequently embedded in paraffin. And then they were cut into 5 μm serial slices. Next, slices were dewaxed and rehydrated sequentially in xylene and a diminishing gradient of alcohol. Then hematoxylin and eosin staining solutions were respectively used to stain the tissue slices and neutral gum was used to seal the sheet. Finally, images were acquired using a 3DHISTECH DX 12 slide scanner (3DHISTECH, Jinan, China).

### Immunohistochemistry

Pre-treated tissue wax blocks were dewaxed and rehydrated. 3% H_2_O_2_ was employed to quench the endogenous peroxidase and sodium citrate buffer at pH 6.0 and a microwave remediation method was applied to retrieve the antigen. Next, the previously treated slices were incubated with 5% bovine serum albumin for 30 min at room temperature, followed by overnight incubation with the anti-GPX4 primary antibody (1:200, T56959, Abmart, China) and the anti-Nrf2 primary antibody (1:600, 16396-1-AP, Proteintech, China) at 4 ℃. The next day, the corresponding secondary antibody was incubated for 1 h after three washes with PBS. Finally, a 3,3-diaminobenzidine tetrahydrochloride (DAB) substrate kit (Beyotime Biotechnology, China) was used for the detection of peroxidase reactivity, and the operation steps were accomplished according to the kit instructions. The images were obtained from the 3DHISTECH DX 12 (3DHISTECH, Jinan, China).

### Screening targets in database

Relevant targets in the GeneCards database [26] were searched using the keywords “premature ovarian failure”, “cisplatin”, “ferroptosis” and “endometrial stem cells”. Targets were included by the following conditions: category as protein coding, gift value > average, and relevance score > average. Lastly, the intersection of targets was acquired.

### Statistical analysis

Data from at least three independent experiments were put into analysis and presented in the form of mean ± standard deviation. All data were statistically analyzed using unpaired student’s t-test (two groups) and one-way ANOVA (multiple groups) with the GraphPad Prism 8 software. A p-value less than 0.05 was considered statistically different.

## Results

### Cisplatin induced ferroptosis in KGN cells

**Fig. 1:**
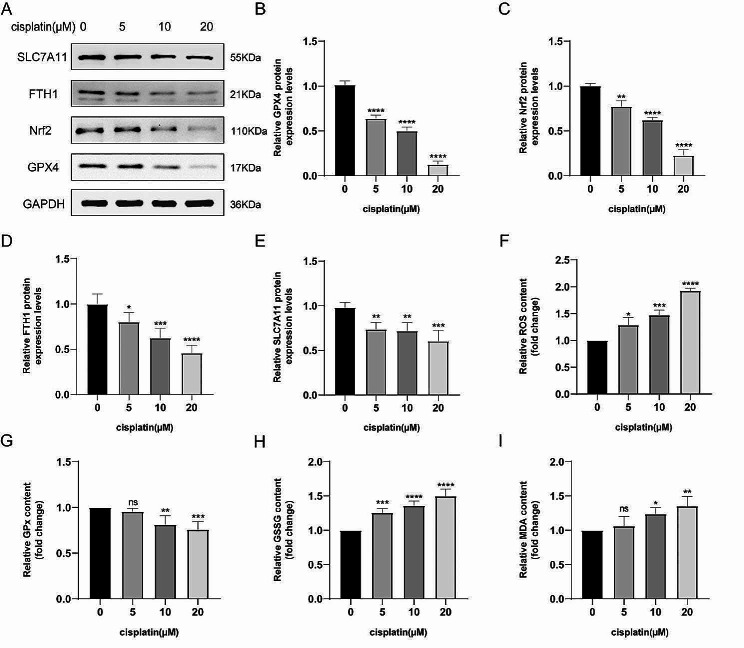
Ferroptosis occurred in cisplatin-injured KGN cells. **A-E** The expression levels of ferroptosis‑related proteins with different concentrations of cisplatin were measured by Western blotting. **F-I** Relative expressions of lipid ROS (**F**), GPx (**G**), GSSG (**H**) and lipid peroxidation MDA (**I**) were assessed in KGN cells treated with gradient concentrations of cisplatin for 24 h. Data were expressed as mean ± SEM from no less than three independent experiments ( *P < 0.05, **P < 0.01, ***P < 0.001, ****P < 0.0001, and ns indicates no statistical significance)

**Fig. 2:**
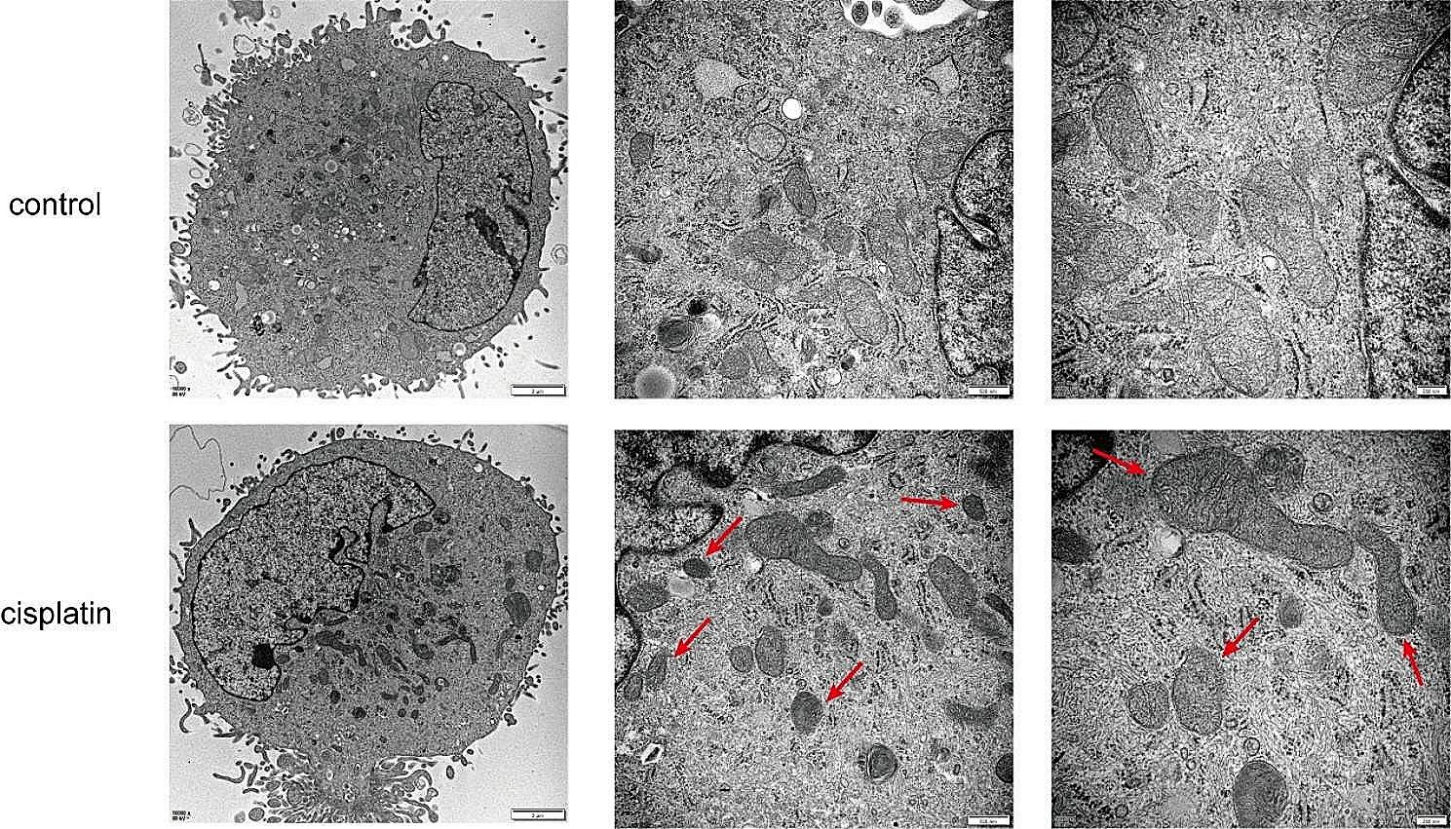
Mitochondrial morphology in cisplatin-injured KGN cells. Red arrows referred to reduced mitochondrial volume, increased mitochondrial density and ruptured outer membrane. The KGN cells were treated for 24 h with 10 µM cisplatin as the cisplatin group. The magnifications of these images are respectively 10000X, 30000X and 50000X. And the scale bars represent 2 μm, 500 nm and 200 nm, respectively

To examine whether cisplatin could induce ferroptosis in KGN, cells were first treated with various concentrations of cisplatin (0, 5, 10, 20 µM). Western blotting was employed to detect the expression levels of ferroptosis-related proteins. Results showed that ferroptosis marker protein GPX4 was down-regulated in a dose-dependent manner (Fig. 1A-B). The same trends can also be observed in the expression levels of Nrf2, FTH1 and SLC7A11 (Fig. 1A and C-E). Then, ROS as one of the typical features of ferroptosis was investigated. Results showed that ROS content was significantly increased after cisplatin treatment compared with that in the control group (Fig. 1F). Similarly, cisplatin also improved the levels of lipid peroxidation MDA and oxidized GSSG, while the levels of GPx in cisplatin-injured KGN cells were reduced (Fig. 1G-I). To further verify that cisplatin could induce ferroptosis in KGN cells, we used electron microscopy to observe the ultrastructure of mitochondria after cisplatin treatment and the characteristic mitochondrial changes were found. The structure of mitochondria in cisplatin-injured KGN cells became smaller and more electrondense compared to the control group, the membrane density was increased and the mitochondrial outer membrane was broken (Fig. 2). These results provide preliminary evidence that cisplatin could induce ferroptosis in KGN cells.

### Fer-1 attenuated the damage of cisplatin on KGN cells

**Fig. 3:**
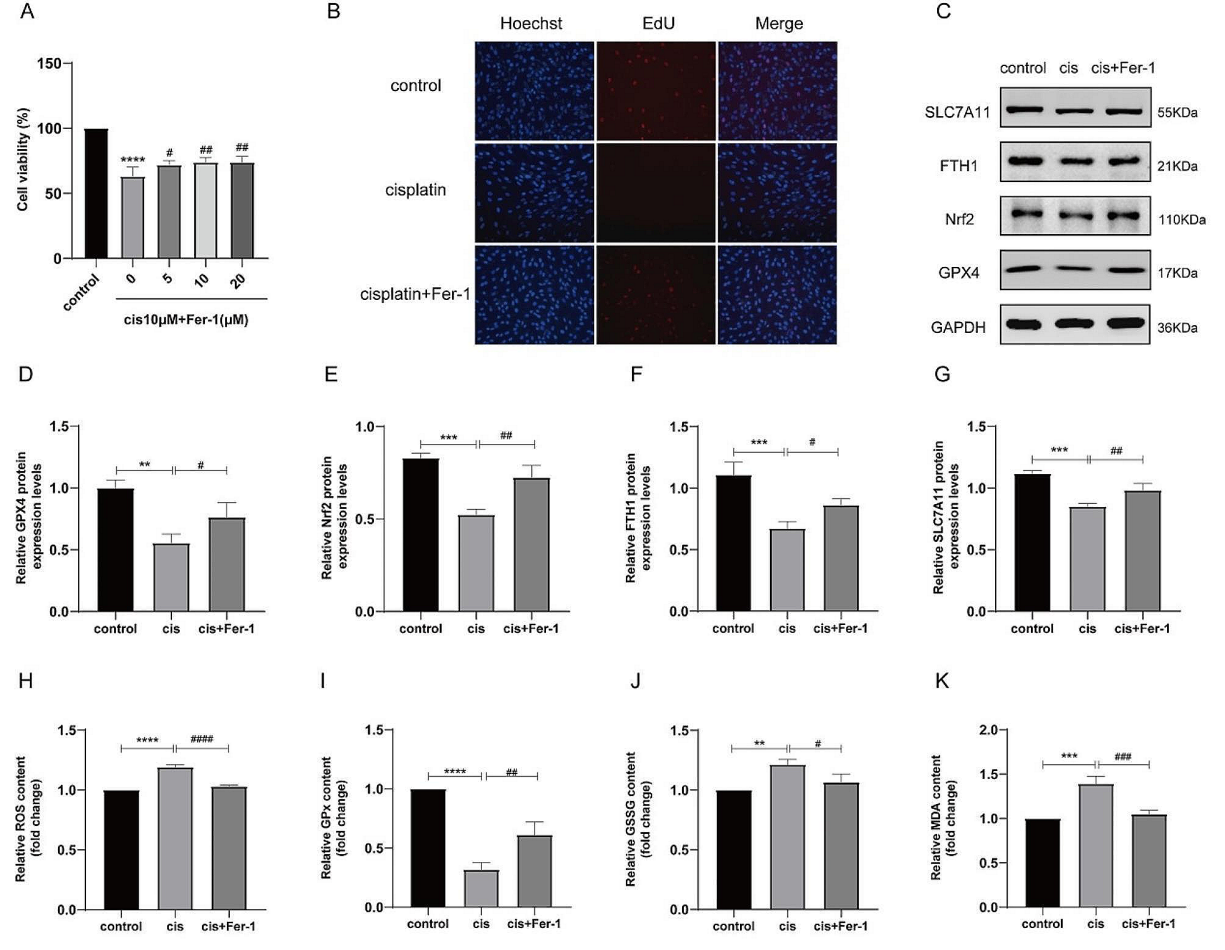
Fer-1 reduced the damage caused by cisplatin in KGN cells by inhibiting ferroptosis. **A** Cell viability was measured by CCK‑8 assay following KGN cells treated with 10 µM cisplatin and different concentrations of Fer-1 for 48 h. Fer-1 recovered KGN cell proliferation ability in a dose-dependent manner. **B** Cell proliferation capacity was evaluated using EdU labeling assay. **C- G** Western blotting exhibited Fer-1 restored the protein expression levels of GPX4, Nrf2, SLC7A11 and FTH1. **H-K** The expression levels of ROS (**H**), GPx (**I**), GSSG (**J**) and MDA (**K**) in cisplatin-injured KGN cells were changed after Fer-1 addition. Data were shown as mean ± SEM from no less than three independent experiments (*P < 0.05, **P < 0.01, ***P < 0.001,****P < 0.0001, * versus control group; ^#^*P* < 0.05, ^##^*P* < 0.01, ^###^*P*< 0.001, ^####^*P* < 0.0001, ^#^ versus cis (10 µM cisplatin) group)

As shown before, we tentatively chose cisplatin with a medium concentration of 10 µM to establish an injury model for subsequent experiments. To further explore the relationship between cisplatin and ferroptosis, Fer-1 as a famous ferroptosis inhibitor with different concentrations (0, 5, 10, 20 µM) was applied to treat injured KGN cells. CCK-8 assay illustrated Fer-1 could restore the reduction of cell viability caused by cisplatin treatment in a dose-dependent manner, reaching obvious significance at 10 and 20 µM (Fig. 3A). 10 µM Fer-1 was ultimately chosen for subsequent experiments. Consistent with the CCK-8 result, EdU assays also displayed that Fer-1 significantly restored the proliferation ability of injured cells (Fig. 3B). Western blotting analysis presented Fer-1 partly increased the expression levels of FTH1, SLC7A11, GPX4 and Nrf2 compared to cisplatin treatment alone (Fig. 3C and G). Besides, KGN cells incubated with Fer-1 and cisplatin displayed substantially decreased ROS, GSSG and MDA levels while increased GPx levels compared with those in the cisplatin alone group (Fig. 3H and K). The above results reexamine that cisplatin could induce ferroptosis in KGN cells and demonstrate that Fer-1 can potentially protect KGN cells from cisplatin-induced damage by inhibiting ferroptosis.

**Fig. 4:**
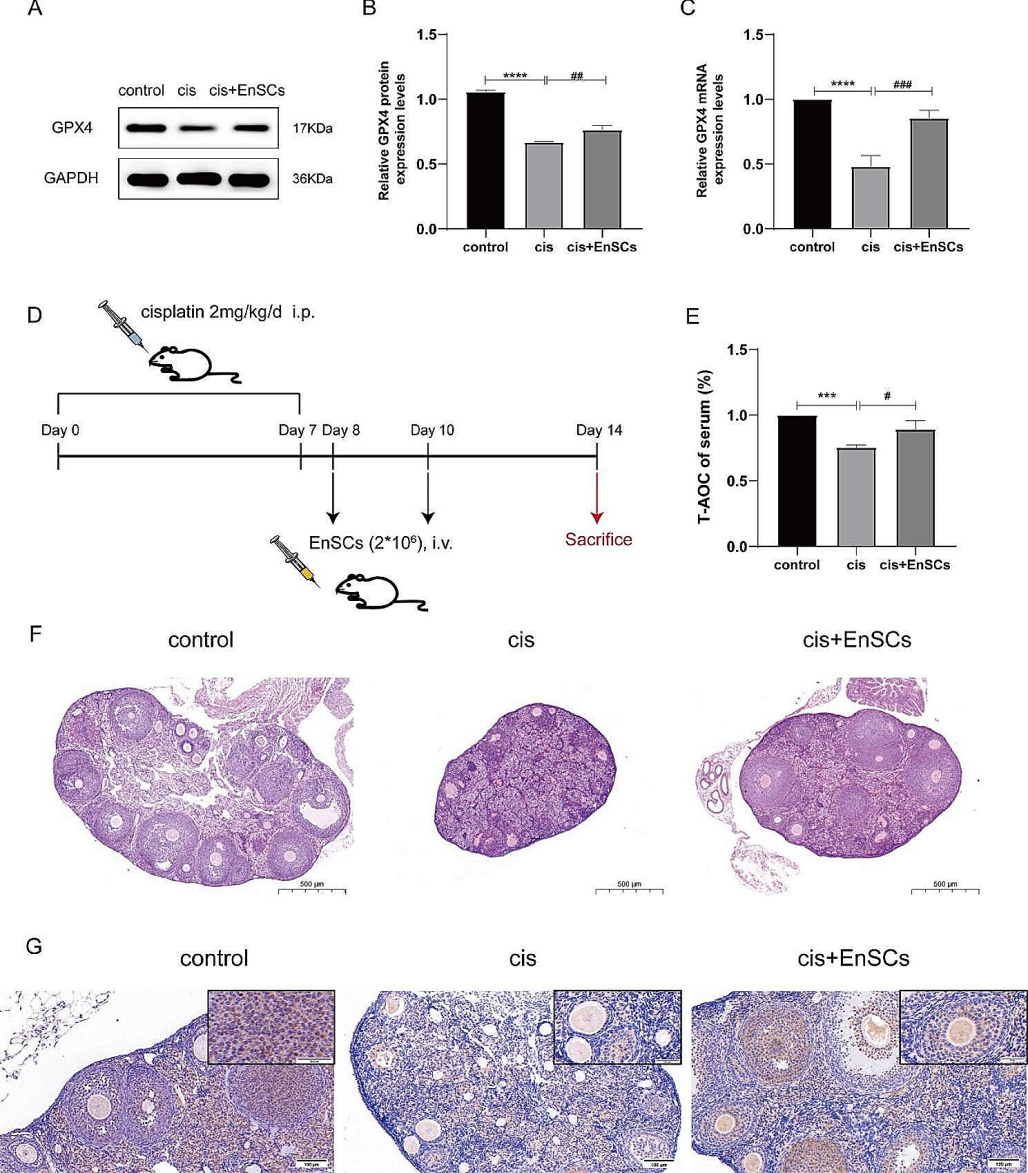
EnSCs reversed cisplatin-induced ferroptosis in vivo and in vitro. **A-B** Western blotting was performed to detect the GPX4 protein expression levels, which were quantified. **C** qRT-PCR was conducted to assess the mRNA expression levels of GPX4. **D** Animal models establishment process. **E** Measurement of serum total antioxidant capacity in mice. **F** Ovarian morphology and follicle numbers were presented by H & E staining. **G** GPX4 expression levels in ovarian tissue were displayed by immunohistochemistry (*n* = 5 mice per group). Scale bars in the HE staining represent 500 μm, while in the IHC staining represent 100 μm. The results were expressed as mean ± SEM (*P < 0.05, **P < 0.01, ***P < 0.001, ****P < 0.0001, * versus control group; ^#^*P* < 0.05, ^##^*P* < 0.01, ^###^*P* < 0.001, ^####^*P* < 0.0001, ^#^ versus cis (10 µM cisplatin) group)

### EnSCs treatment reversed cisplatin-induced ferroptosis both in vivo and in vitro

EnSCs have aroused widespread attention for their potential to alleviate POF. Our previous experiments have confirmed EnSCs can reduce apoptosis levels and recover proliferative levels to repair injured granulosa cells [25]. Herein we tried to figure out whether ferroptosis inhibition also participates in the repair process of EnSCs. As expected, we found the expression levels of GPX4, the hallmark of ferroptosis, have been statistically significantly restored compared to the cisplatin alone group at both the protein levels (Fig. 4A-B) and the mRNA levels (Fig. 4C) in the cell coculture model. Furthermore, we established the animal models following our previous study [7, 25] (Fig. 4D). We found the expression levels of GPX4 in ovary tissue were decreased in the POF group, while it was recovered in the EnSCs transplantation group, which was consistent with in vitro experiments (Fig. 4G). Then, serum was collected and the total antioxidant capacity was detected. Results presented that the total antioxidant capacity of mouse serum was elevated after EnSCs transplantation (Fig. 4E). Besides, HE staining illustrated that the ovarian morphology and follicle morphology and numbers in the EnSCs treatment group were much better than the cisplatin injection group (Fig. 4F). Meanwhile, Fer-1 was used as the positive control. We found Fer-1 could also enhance the total antioxidant capacity of mouse serum (Fig. S1B). Rather normal ovarian structure and follicular shape can also be observed in the Fer-1 group than in the DMSO control group (Fig. S1C). Taken together, these results confirmed treatment with EnSCs ameliorated ovarian function in POF mice and supported the hypothesis that EnSCs treatment is a promising candidate for the treatment of POF by mitigating cisplatin-induced ferroptosis.

### The repair effect of EnSCs may depend on Nrf2

**Fig. 5:**
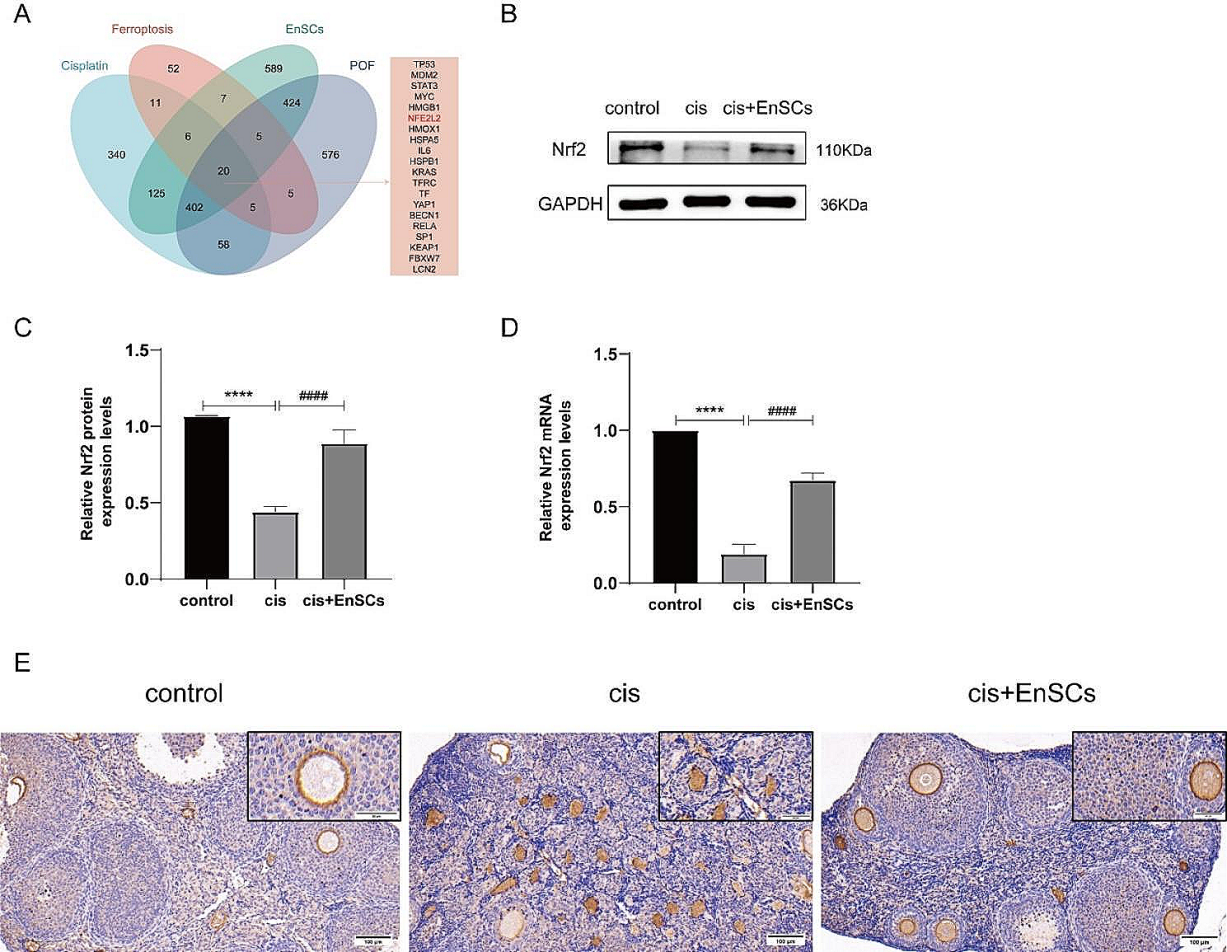
Nrf2 may be a key driver for EnSCs to inhibit ferroptosis and repair damage. **A** Combinatorial analysis of the related genes about POF, cisplatin, ferroptosis and EnSCs uncovered 20 common genes, among which was Nrf2. **B-C** Nrf2 protein expression levels were detected by Western blotting. **D** Nrf2 mRNA expression levels were detected by qRT-PCR. **E** Nrf2 protein expression levels in ovarian tissue were detected by immunohistochemistry. Scale bars in the IHC staining represent 100 μm. The results were expressed as mean ± SEM (****P < 0.0001, * versus control group; ^####^*P* < 0.0001, ^#^ versus cis (10 µM cisplatin) group)

Next, we tried to reason out the underlying mechanisms of EnSCs treatment. Data collected from the GeneCards database provided a theoretical basis that Nrf2 is a critical regulatory target (Fig. 5A, Table S2). Then, Western blotting (Fig. 5B-C), qRT-PCR (Fig. 5D), and IHC (Fig. 5E) results exhibited that EnSCs restore the expression of Nrf2 not only in injured granulosa cells but also in POF mice. These findings illustrated that Nrf2 may have the ability to explain, at least in part, the mechanism by which EnSCs improve POF ovarian function.

### ML385 abolished the repair effect of EnSCs on cisplatin-injured KGN cells

**Fig. 6:**
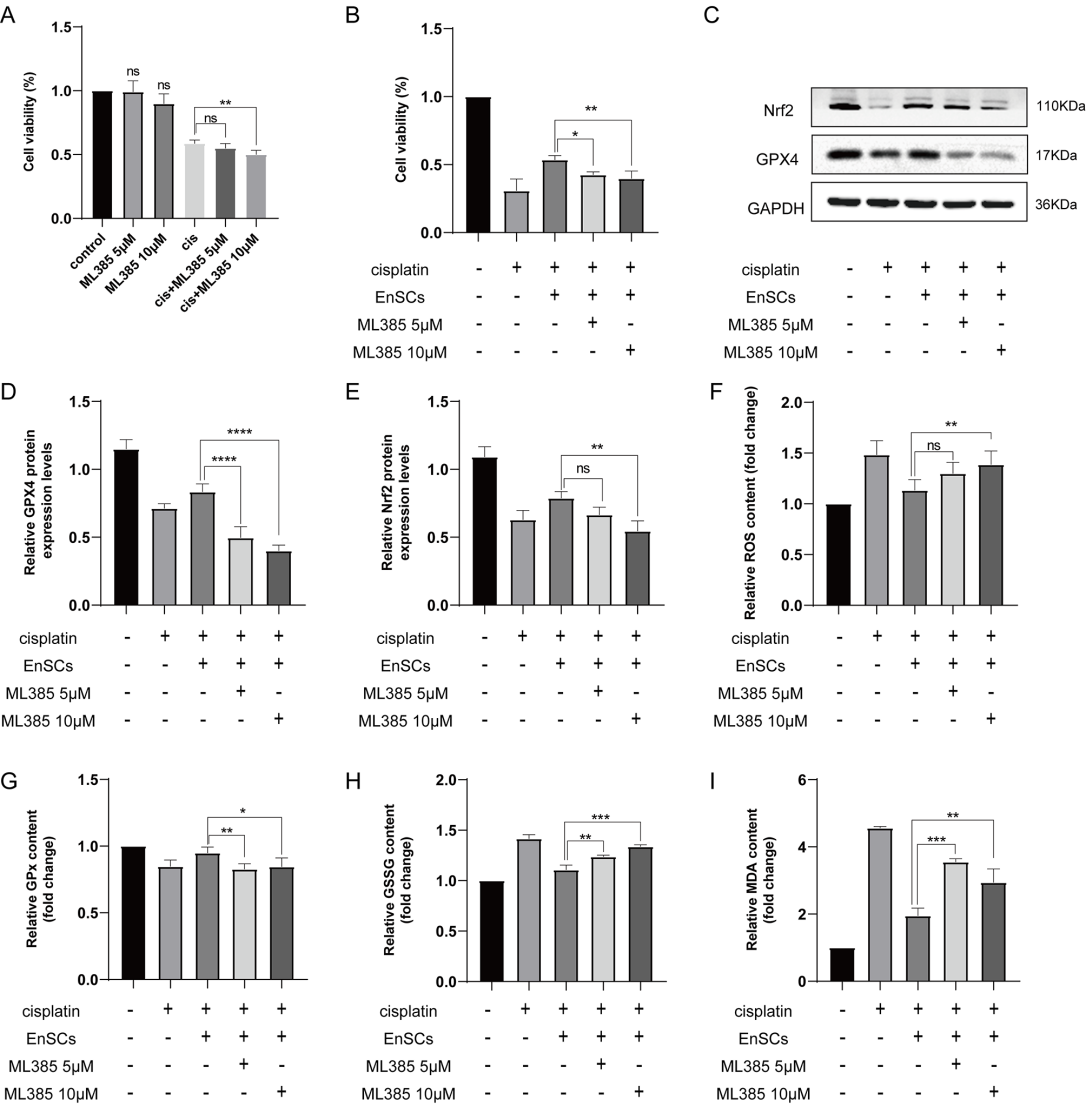
The Nrf2-specific blocker ML385 abolished the protective effect of EnSCs on cisplatin-injured KGN cells. **A-B** Evaluation of cell proliferation ability. The KGN cells were treated for 48 h with ML385 (5 µM or 10 µM) with or without 10 µM cisplatin. Condition medium obtained from the EnSCs was used in the EnSCs group to restore injured KGN cells’ proliferation. Different groups were incubated with condition medium and ML385 (5 µM or 10 µM) for 72 h in accordance with need. **C-E** Measurement of the protein expression levels of GPX4 and Nrf2. **F-I** The relative contents of ROS (**F**), GPx (**G**), GSSG (**H**), and MDA (**I**) were assessed in the coculture model with ML385 addition. The results were shown as mean ± SEM. (*P < 0.05, **P < 0.01, ***P < 0.001, ns indicates no statistical significance)

To elucidate whether the restored expression of Nrf2 caused by EnSCs was responsible for the restorative effects by inhibiting ferroptosis, the Nrf2-specific inhibitor ML385 was used to investigate it in the cell coculture model. No obvious influence on cell viability was found when ML385 treated KGN cells alone, but adding ML385 based on cisplatin aggravated the damage to KGN cells (Fig. 6A). Then, we found ML385 addition abolished the increased cell viability caused by EnSCs in cisplatin-injured KGN cells (Fig. 6B). Similarly, ML385 partially abrogated the increased protein expression levels of GPX4 and Nrf2 restored by EnSCs (Fig. 6C-E). Furthermore, ML385 markedly decreased the levels of GPx, the core enzyme that regulates the antioxidant system, while increasing the ferroptosis indicators ROS, GSSG and lipid peroxidation MDA levels (Fig. 6F-I). These results demonstrated that Nrf2 inhibition still made KGN cells more susceptible to ferroptosis even after EnSCs treatment. Therefore, the elevation of Nrf2 caused by EnSCs may contribute to its inhibition of ferroptosis and thus play a repair role.

## Discussion

POF as a common and complex gynecological endocrine disease is gradually familiar to us. Unfortunately, POF remains an unsolved problem that lacks specific and effective treatment now. Many clinically used methods such as hormone replacement therapy (HRT) [27] and cryotherapy [28] do not get satisfactory results because they cannot fundamentally repair ovarian damage, so POF urgently needs better treatments. Attention is converted to stem cell therapy due to its rapid development in recent years. In 2018, Dai Jianwu and Sun Haixiang’s team found that collagen-loaded umbilical cord mesenchymal stem cells could activate primordial follicles in vitro by phosphorylating FOXO3a and FOXO1, and also increased estradiol concentration and the number of antral follicles in vivo to save the overall function of the ovaries of POF patients with long-term infertility [29]. Moreover, Zafardoust et al. discovered the transplantation of autologous menstrual blood derived-mesenchymal stromal cells had therapeutic efficacy that included improving ovarian function and restoring menstrual cycles for some POF patients [30]. Various stem cells have been widely applied to treat POF and acquired some therapeutic effects in clinical trials. Regrettably, the majority of practical applications are still not ideal, and the specific repair mechanism is not yet clear. For better clinical applications of stem cell therapy in POF, the first and critical step is to fully understand the related mechanisms in treating POF to ensure its safety and efficacy. Hence, in this situation, new experiments are urgently needed to carry out and explore.

Many researches have validated that the occurrence of POF is tightly associated with the apoptosis of granulosa cells [25, 31, 32]. And it was reported that autophagy is also involved [33–35]. However, little is known about ferroptosis, another prevalent form of programmed cell death, whether participates in the pathogenesis of POF. Ferroptosis has been acknowledged to take part in the pathogenesis of many diseases such as cancers [36–38] and neurodegenerative diseases [39, 40], but there are few reports in the field of POF. Geng et al. reported electroacupuncture can efficiently suppress ovarian oxidative stress and Fe^2+^ accumulation in POF mice, which offers a possibility that ferroptosis may be linked to POF [17]. There are even more straightforward experiments to state ferroptosis is associated with chemotherapy-induced POF. Research results confirmed cisplatin induces ovarian damage by promoting ferroptosis [41]. Another study showed ferroptosis mediates cisplatin-induced ovarian fibrosis thus leading to POF in rats [16]. We further confirm that ferroptosis is involved in the occurrence and development of POF. Nevertheless, there is still much room to study and explore the relationship between POF and ferroptosis.

At the beginning of this experiment, we treated KGN cells with gradient concentrations of cisplatin to establish injured models. Cisplatin has been authenticated to induce the occurrence of ferroptosis in some cancer cells [42] and renal tubular epithelial cells [43, 44]. We first proved cisplatin can induce ferroptosis in KGN cells in a dose-dependent manner by detecting the protein expression levels of ferroptosis-related molecules such as GPX4, Nrf2, FTH1 and SLC7A11. However, the downregulation of FTH1 seems to be contradictory to some findings [45]. FTH1 is an important component of transferrin, which can reduce intracellular free iron by exerting iron storage function. Therefore, when cisplatin-induced ferroptosis occurs, FTH1 expression should be decreased in theory, which is consistent with many experimental results [46, 47]. And related ferroptosis indicators such as ROS, glutathione peroxidase GPx, oxidative stress GSSG and lipid peroxidation MDA were also examined to indirectly determine the degree of ferroptosis.

For the convenience of subsequent experiments, we chose 10 µM as one moderate cisplatin concentration. The purpose of choosing this concentration is to ensure that the notable difference will not be neglected due to too little damage and that the subsequent repair will not work due to too much damage. Besides, if the concentration is too high and the cells are almost completely dead, some indicators of ferroptosis such as ROS which need to be conducted in living cells are undetectable.

To further verify the existence of ferroptosis in injured KGN cells, we used transmission electron microscopy to observe mitochondrial morphology. Images displayed typical morphological characteristics of ferroptosis, including mitochondrial volume reduction, double membrane density increase and mitochondrial outer membrane rupture, which is consistent with previous experiments [48]. Subsequently, ferroptosis inhibitor ferrostatin-1 was introduced in the study. We found Fer-1 reduced the damage caused by cisplatin in KGN cells by inhibiting ferroptosis to further confirm cisplatin can induce ferroptosis in KGN cells.

Next, our previous experiments have confirmed that EnSCs can mitigate cisplatin-induced granulosa cell damage and alleviate ovarian damage in POF mice [25]. Herein, the same repair effects have been received again. Intriguingly, we discovered EnSCs can upregulate the expression of ferroptosis marker protein GPX4 both in vivo and in vitro. And we found that EnSCs play a similar role as Fer-1 by detecting the ferroptosis-relevant indicators again. Results suggested EnSCs treatment reversed cisplatin-induced ferroptosis both in vivo and in vitro. Although our previous study has clarified EnSCs can exert repair effects by inhibiting apoptosis of granulosa cells [25], it is not contradictory to the finding of this study. Because whether cells or organisms, either of the two is an organic combination, not a single repetitive programming. There are multiple of researches reporting that ferroptosis is coordinated with apoptosis to take part in the development and treatment of diseases [49–51]. Cisplatin may also induce ferroptosis while inducing cell apoptosis, as does EnSCs repair. The therapeutic effect of EnSCs is not upon a single factor but a sophisticated biological regulation. However, whether the relationship between ferroptosis and apoptosis or other death modes is complementary or independent needs further exploration.

To decipher the possible mechanism accounting for the repair effect of EnSCs, we used the GeneCards database to screen out 20 targets, and we attached more attention to Nrf2 which is a well-known transcription factor to mediate the antioxidant pathway. Nrf2 is generally acknowledged to play a central role in upregulating anti-ferroptosis defense [52, 53]. Previous studies have already proven that Nrf2 participated in ovarian protection in many POF models [21, 54]. And stem cells can also govern ferroptosis sensitivity by adjusting Nrf2 expression in many diseases [55–57]. Therefore, we paid particular attention to the expression of Nrf2 in the process of our experiment, and we considered that Nrf2 is an important regulatory point for EnSCs to play a restorative role. To examine our hypothesis, ML385, the specific inhibitor of Nrf2 [58], was introduced in our experiment. Nevertheless, experimental results suggested that the change in cell viability was not significant when ML385 and cisplatin acted simultaneously. It may be that the stronger damaging effect of cisplatin on cells has largely depleted intracellular Nrf2 so the inhibitive ability of ML385 was not prominent. But ML385 significantly altered the cell viability that was recovered by EnSCs. As expected, ML385 restored the expression levels of ferroptosis-related proteins and partly abolished the protective effect of EnSCs on cisplatin-injured KGN cells. Therefore, there is a reasonable presumption that EnSCs inhibit ferroptosis achieving repair effects through upregulating Nrf2 expression.

Regretfully, we have known Nrf2 played a decisive role in the repair function of EnSCs, but its downstream targets and specific pathways were not explored in this experiment. Previous studies have shown that Nrf2 can directly induce SLC7A11 expression at the transcriptional level by binding to its promoter to increase cysteine supply [59–61]. Naringenin and melatonin have also been demonstrated to respectively alleviate myocardial and renal ischemia/reperfusion injury by regulating Nrf2/SLC7A11/GPX4 axis to inhibit ferroptosis [62–64]. Therefore, we speculate EnSCs mitigated cisplatin-induced damages probably also through the same pathway. This can be explored in our subsequent experiments.

In this study, we treated POF mice by tail vein injection of EnSCs. This cell therapy using mesenchymal stem cells is being widely explored in basic experiments and clinical trials. However, its safety and efficacy remain to be further evaluated. Although stem cells from multiple sources have achieved significant therapeutic effects in POF animal models [7, 65–67], there is no uniform standard for the method of stem cell extraction and the number and method of transplantation, so the safety and efficacy of its application cannot be guaranteed. Besides, to ensure the efficacy of stem cell therapy, stem cells are required in vitro expansion to a certain number, while in vitro-treated cells may change the inherent biological properties of the stem cells over time and culture conditions, leading to unexpected differentiation results and deleterious implantation after stem cell transplantation. In particular, stem cells have strong adhesive properties and can easily cluster in blood vessels, thus forming life-threatening embolisms. Multiple injections over a long period will probably increase the chances of embolisms and thus reduce their safety. Although stem cell therapy is currently achieving good efficacy in a variety of animal models of disease, it remains too early to be confident that stem cell therapy is very safe.

It is undeniable that there are a lot of limitations in this study. Firstly, we do not know exactly which component of EnSCs plays a key role in the repair process due to the limitation of experimental conditions. The cost of stem cell treatment is extremely expensive and the therapeutic effects vary from person to person, so on the foundation of our experiment, cell-free therapy with EnSCs can be further advanced and other ferroptosis-related compounds are also worthy of application and development to treat POF. Moreover, EnSCs can play a role in repairing damaged granulosa cells by inhibiting both apoptosis and ferroptosis, so we cannot distinguish which part is more crucial. Or how they coordinately interact with each other in the process. We initially found recovery of gene expression and follicular morphology, but there is a lack of further analysis of maturation and hormone secretion of follicles in treated ovaries. Admittedly, our findings are weak, but the work will continue. Despite these limitations, we are still able to illustrate that cisplatin triggers ferroptosis in granulosa cells and there is a close relationship between ferroptosis and POF. Meanwhile, our study proves the ability of EnSCs to repair ovarian damage caused by cisplatin by inhibiting ferroptosis in granulosa cells.

## Conclusion

**Fig. 7:**
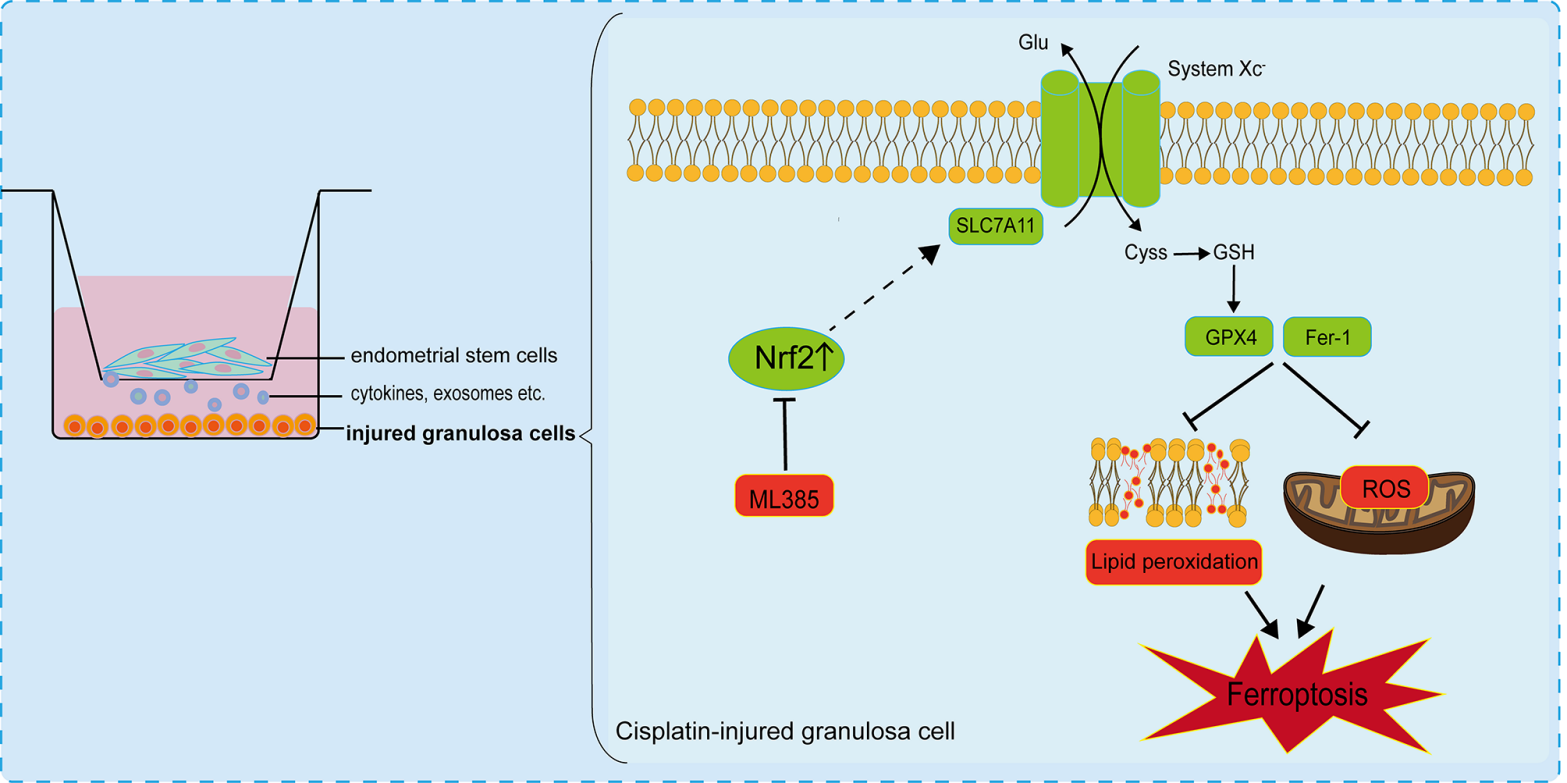
Schematic model depicting the repair mechanism of EnSCs against cisplatin-induced ferroptosis in granulosa cells. As shown in the figure, endometrial stem cells can upregulate Nrf2 expression in injured granulosa cells, while Nrf2 may further prompt the SLC7A11-GPX4 axis to inhibit ferroptosis thus taking a protective role in cisplatin-injured granulosa cells, which can be blocked by ML385. Meanwhile, EnSCs exert a similar repair effect as Fer-1 to inhibit the production of reactive oxygen ROS and lipid peroxidation. In conclusion, endometrial stem cells can inhibit cisplatin-induced ferroptosis of granulosa cells through upregulation of Nrf2 expression to achieve repair effects Additional file 1

In summary, as demonstrated in Fig. 7, our study suggested that cisplatin could induce ferroptosis in granulosa cells and EnSCs effectively inhibit cisplatin-induced ferroptosis to alleviate damages. Furthermore, the repair effect of EnSCs can be partially blocked by ML385 which manifests Nrf2 plays a vital role in the repair process. Ferroptosis has been poorly studied in POF, our findings provide insight into ferroptosis and POF and complement the mechanism of stem cell therapy for POF, which may be conducive to further developing POF treatment.

### Electronic supplementary material

Below is the link to the electronic supplementary material.

Supplementary Material 1

Supplementary Material 2

Supplementary Material 3

Supplementary Material 4

## Data Availability

All data collected or analyzed in the study are included in this published article and can be acquired from the corresponding author on reasonable request.

## Abbreviations

CCK-8: Cell counting kit-8
Cis: Cisplatin
EdU: 5-ethynyl-2-deoxyuridine
EnSCs: Endometrial stem cells
Fer-1: Ferrostatin-1
FTH1: Ferritin heavy chain 1
GPx: Glutathione peroxidase
GPX4: Glutathione peroxidase 4
GSSG: Oxidized glutathione disulfide
HRT: Hormone replacement therapy
MDA: Malonaldehyde
MSCs: Mesenchymal stem cells
Nrf2: Nuclear factor erythroid-2 related factor 2
POF: Premature ovarian failure
ROS: Reactive oxygen species
SLC7A11: Solute carrier family 7 member 11
T- AOC: Total antioxidant capacity
