# Supplementary material for: Endometrial stem cells alleviate cisplatin-induced ferroptosis of granulosa cells by regulating Nrf2 expression
Source: Reprod Biol Endocrinol. 2024 Apr 11;22:41. doi: 10.1186/s12958-024-01208-8 (PMC11008046; doi:10.1186/s12958-024-01208-8)
Supplement: Supplementary file 1 — Supplementary Material 1 [file 12958_2024_1208_MOESM1_ESM.pdf]

SLC7A11

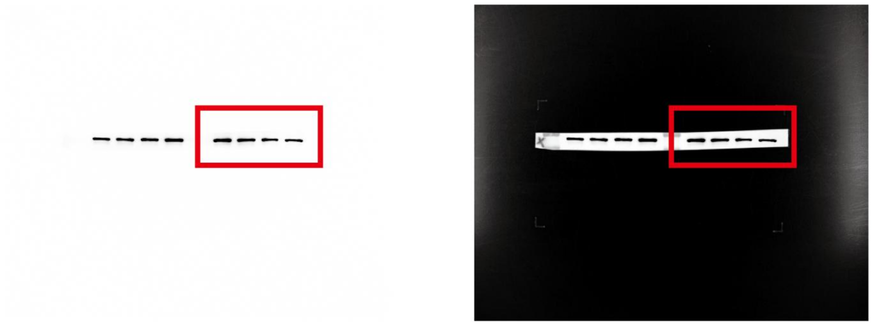

FTH1

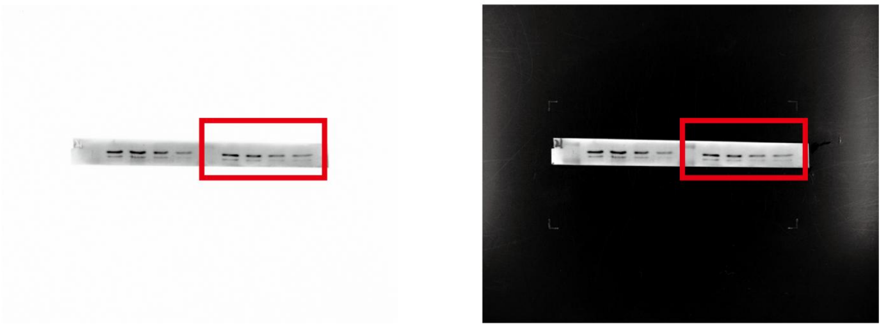

Nrf2

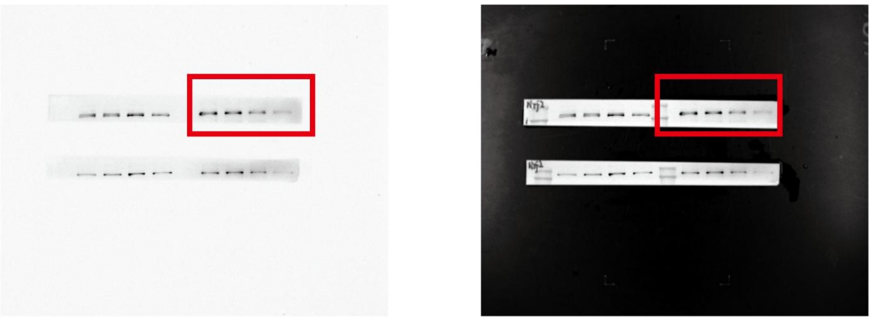

GPX4

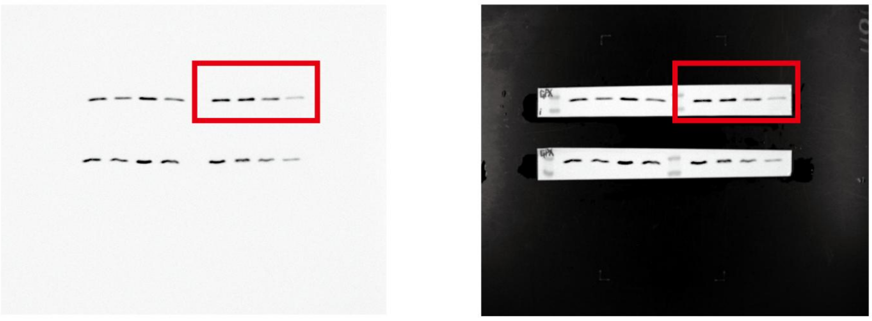

GAPDH

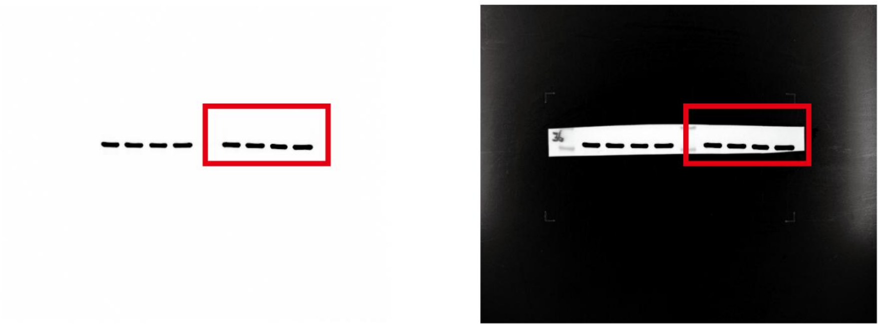

These are the original images of Western blotting from Figure 1. Four samples are shown as one group in the images, and the order of sampling is gradient concentration cisplatin (0, 5, 10, 20 μM). The red frames select the original images used in the manuscript.

SLC7A11

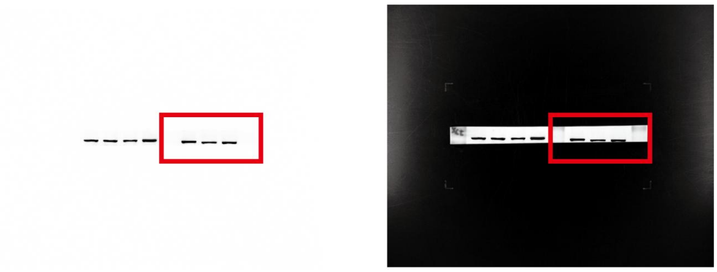

FTH1

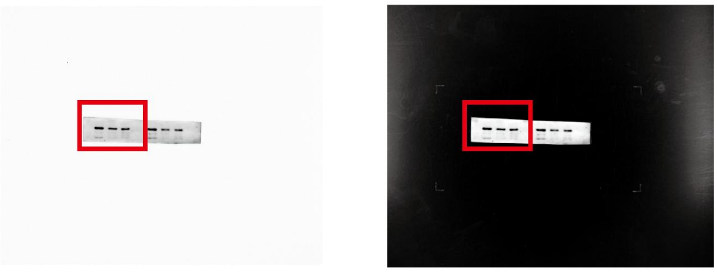

Nrf2

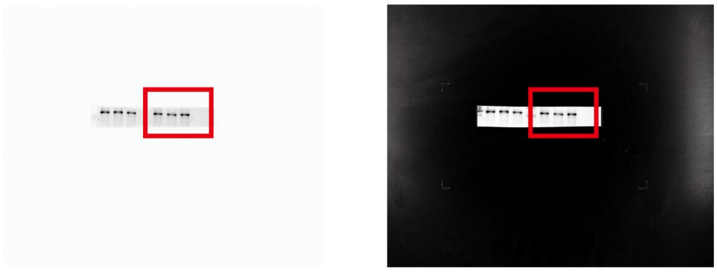

GPX4

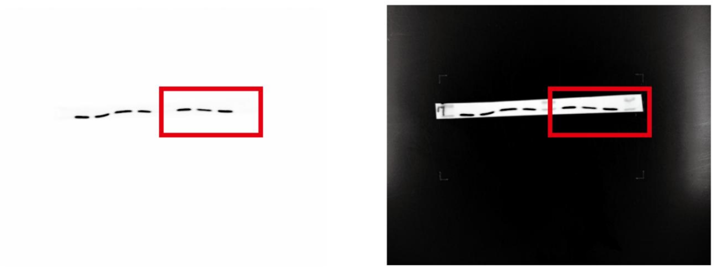

GAPDH

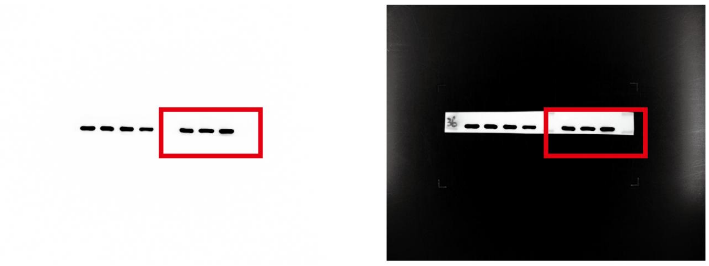

Supplementary
GAPDH for
FTH1 and Nrf2

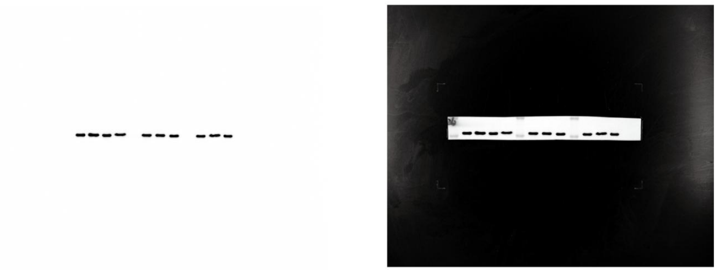

These are the original images of Western blotting from Figure 3. Three samples are shown as one group in the images. The groups are respectively the control group, cisplatin group and cisplatin+Fer-1 group. The red frames select the original images used in the manuscript. Supplementary GAPDH image is exclusive for FTH1 and Nrf2 as an internal reference.

GPX4

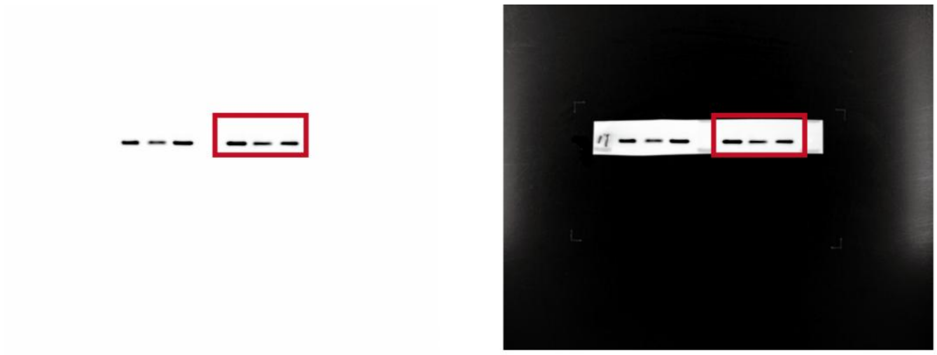

GAPDH

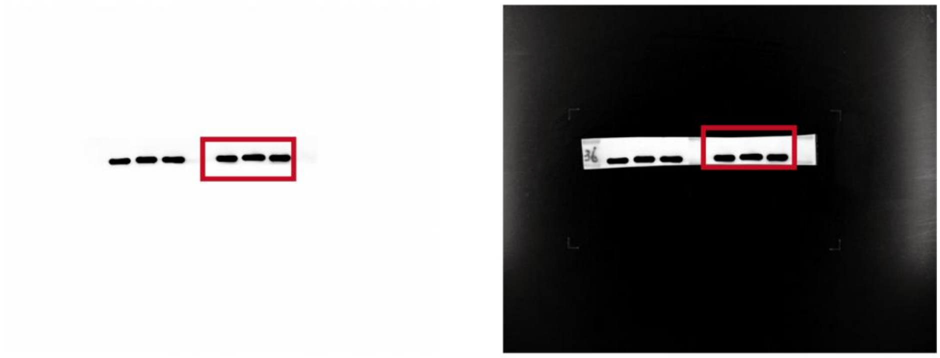

Nrf2

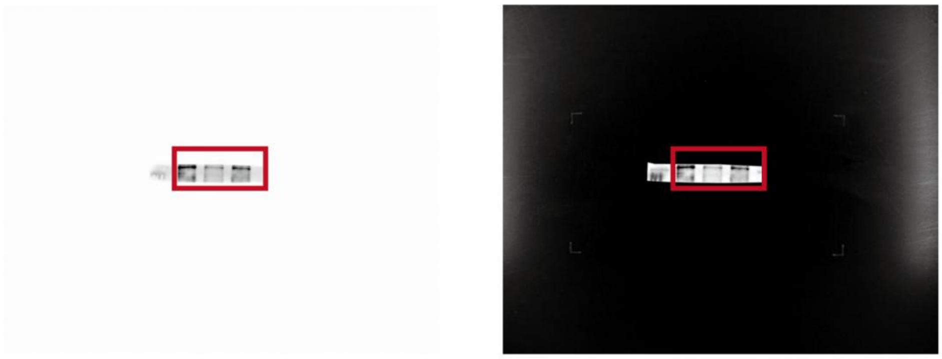

GAPDH

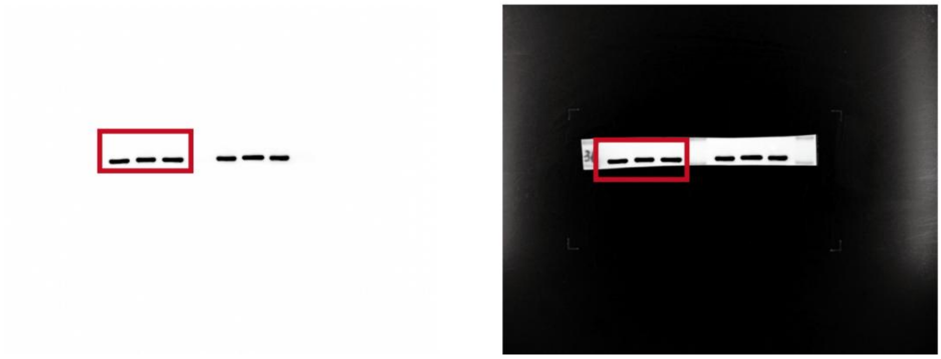

These are the original images of Western blotting from Figure 4 and Figure 5. Three samples are also shown as one group in the images. The groups are respectively the control group, cisplatin group and cisplatin+EnSCs group. The red frames select the original images shown in the manuscript. Parts of some membranes are missing because of cutting for other antibody detection.

Nrf2

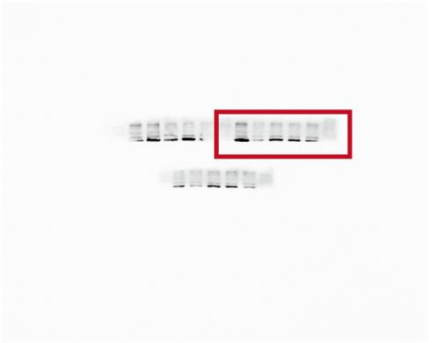

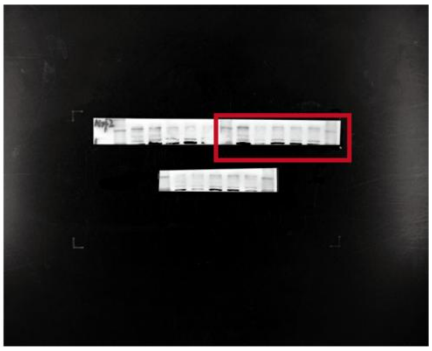

GPX4

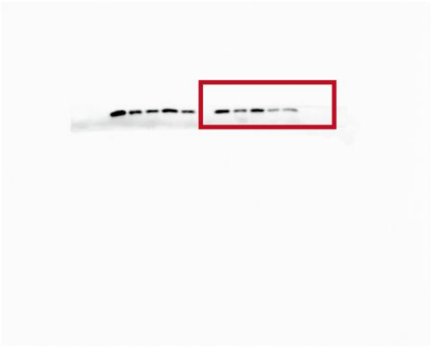

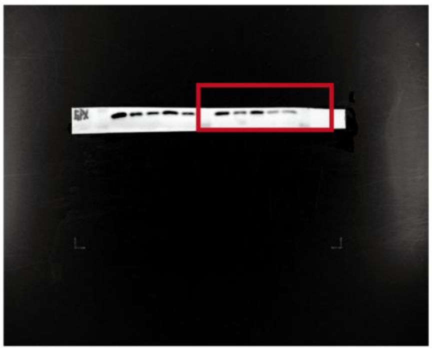

GAPDH

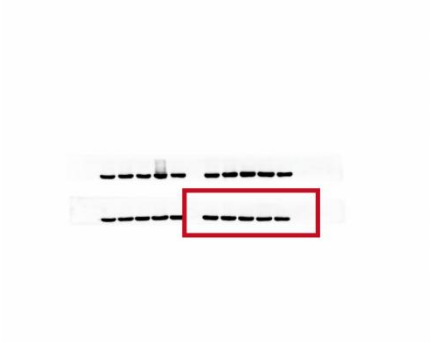

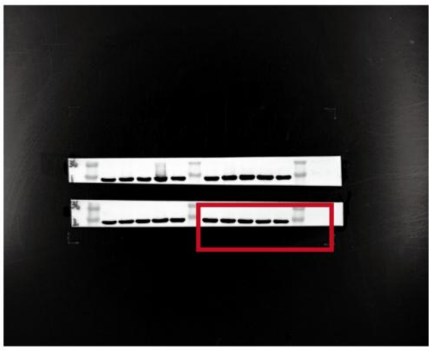

These are the original images of Western blotting from Figure 6. Five samples are presented as one group in the images. The group classification is complex and details are in accordance with the grouping of the manuscript. The red frames select the original images shown in the manuscript.

All images in the manuscript were captured by the chemiluminescent imager (Tanon-5200, China) and edited by the image software Photoshop 2022. In this additional file 3, all images are original and uncropped.
