# Supplementary figures and images for: Endometrial stem cells alleviate cisplatin-induced ferroptosis of granulosa cells by regulating Nrf2 expression
Source: Reprod Biol Endocrinol. 2024 Apr 11;22:41. doi: 10.1186/s12958-024-01208-8 (PMC11008046; doi:10.1186/s12958-024-01208-8)

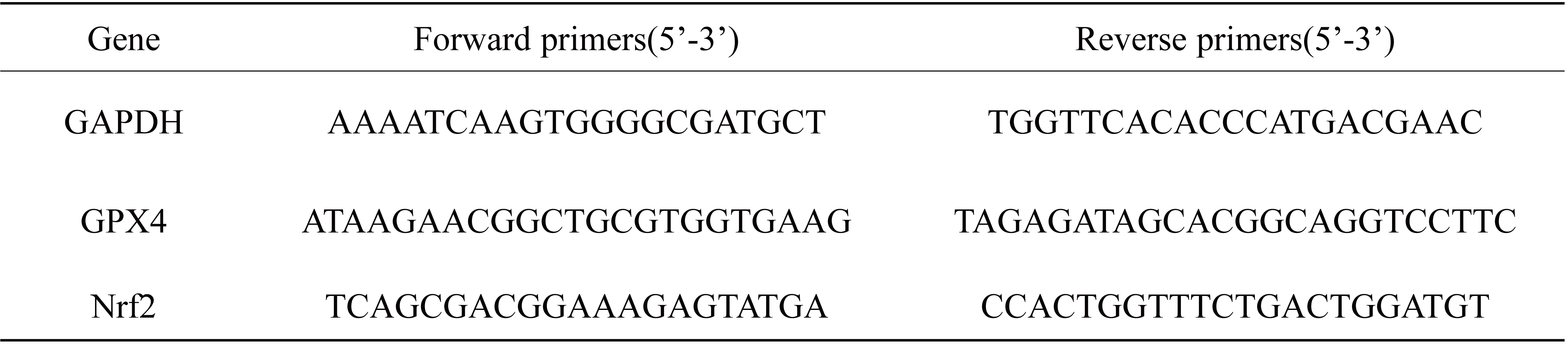

Supplement: Supplementary file 2 — Supplementary Material 2 [file 12958_2024_1208_MOESM2_ESM.tif]

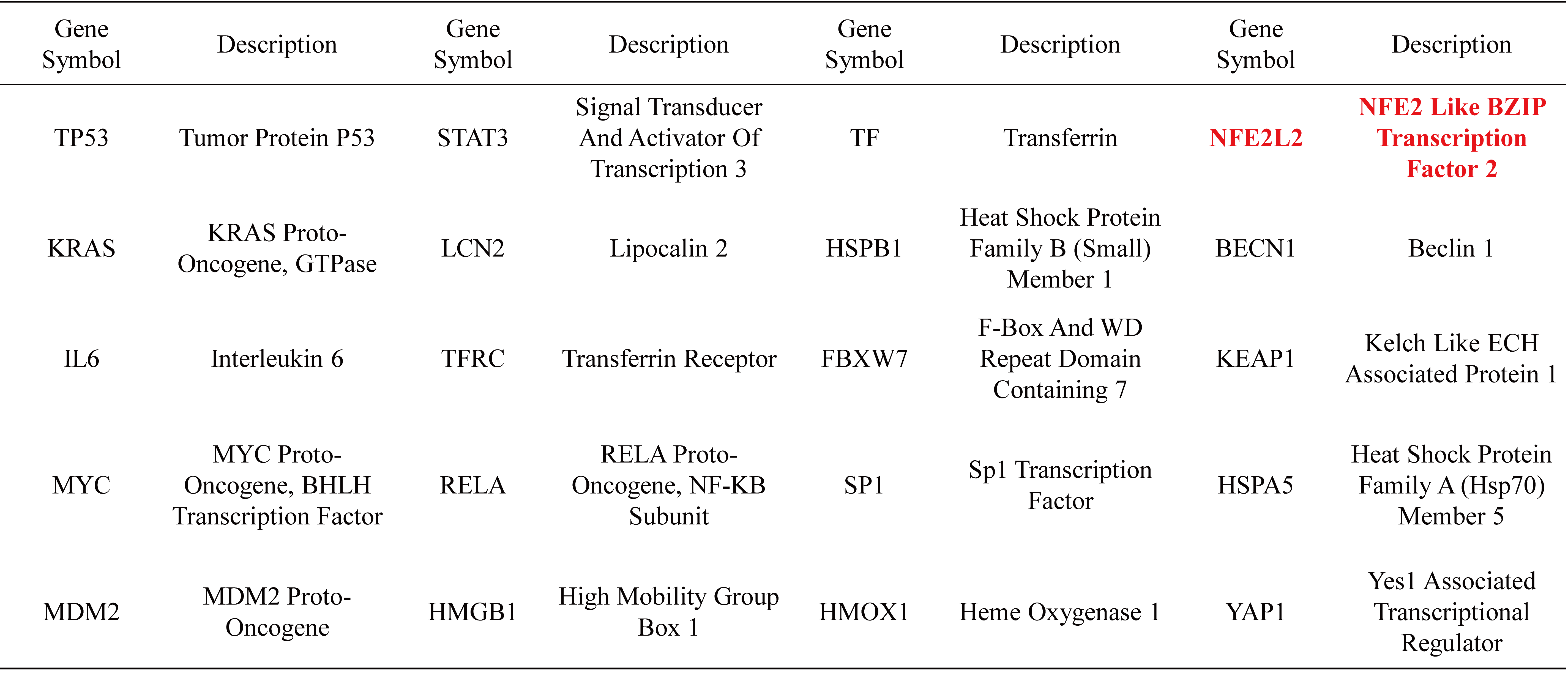

Supplement: Supplementary file 3 — Supplementary Material 3 [file 12958_2024_1208_MOESM3_ESM.tif]

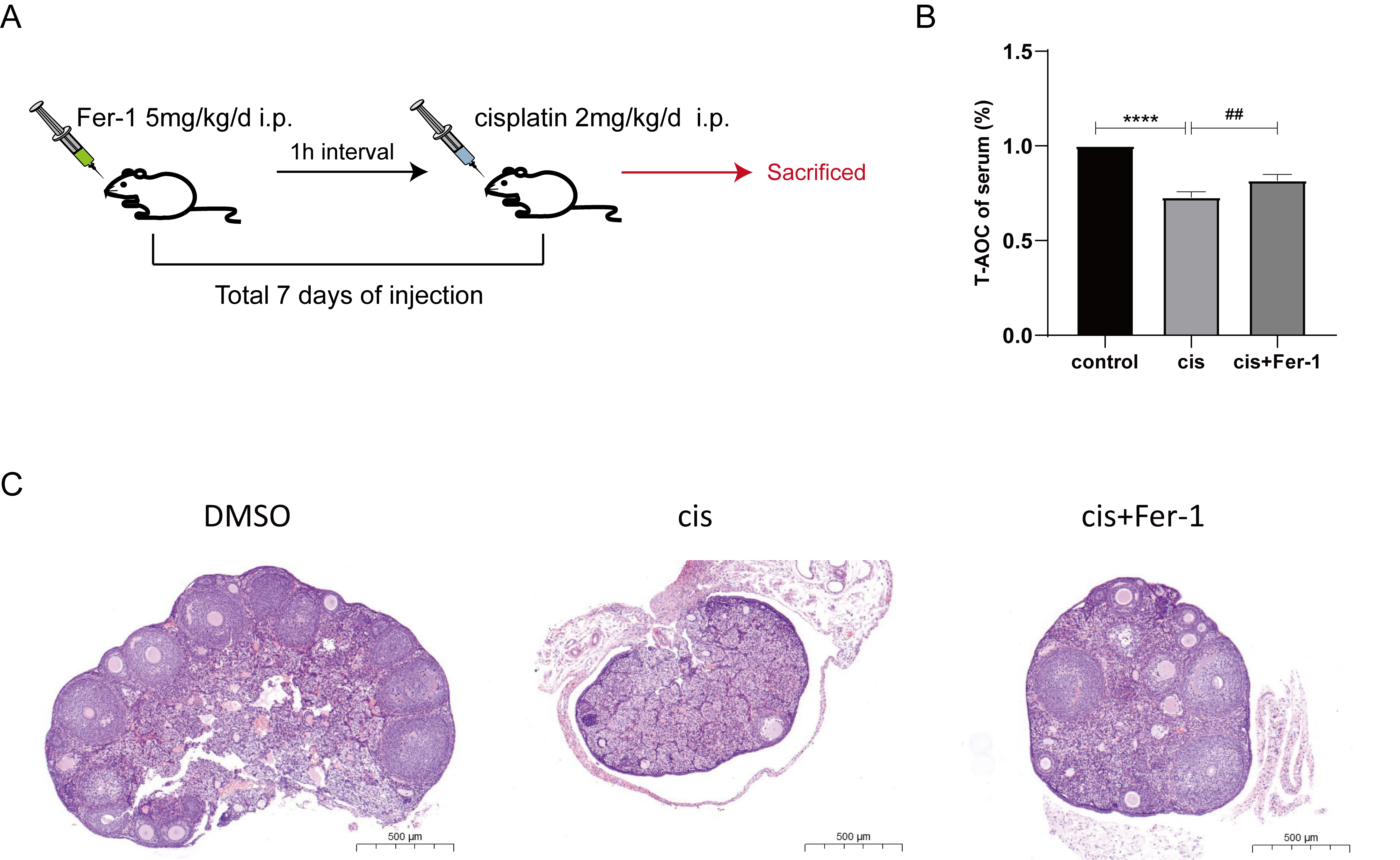

Supplement: Supplementary file 4 — Supplementary Material 4 [file 12958_2024_1208_MOESM4_ESM.tif]
